# Application of Photocatalysis and Sonocatalysis for Treatment of Organic Dye Wastewater and the Synergistic Effect of Ultrasound and Light

**DOI:** 10.3390/molecules28093706

**Published:** 2023-04-25

**Authors:** Guowei Wang, Hefa Cheng

**Affiliations:** MOE Key Laboratory for Earth Surface Processes, College of Urban and Environmental Sciences, Peking University, Beijing 100871, China

**Keywords:** sonocatalysis, sonoluminescence, photocatalysis, organic dye, sonophotocatalysis, synergistic mechanism

## Abstract

Organic dyes play vital roles in the textile industry, while the discharge of organic dye wastewater in the production and utilization of dyes has caused significant damage to the aquatic ecosystem. This review aims to summarize the mechanisms of photocatalysis, sonocatalysis, and sonophotocatalysis in the treatment of organic dye wastewater and the recent advances in catalyst development, with a focus on the synergistic effect of ultrasound and light in the catalytic degradation of organic dyes. The performance of TiO_2_-based catalysts for organic dye degradation in photocatalytic, sonocatalytic, and sonophotocatalytic systems is compared. With significant synergistic effect of ultrasound and light, sonophotocatalysis generally performs much better than sonocatalysis or photocatalysis alone in pollutant degradation, yet it has a much higher energy requirement. Future research directions are proposed to expand the fundamental knowledge on the sonophotocatalysis process and to enhance its practical application in degrading organic dyes in wastewater.

## 1. Introduction

In recent years, the textile industry has played a vital role in the global economy, but it is also a major contributor to environmental pollution, particularly in terms of organic dye wastewater [1,2,3,4,5]. The discharge of organic dye wastewater from textile production and utilization can cause significant damage to the aquatic ecosystem, and therefore, it is imperative to develop effective treatment methods to degrade these pollutants [6,7,8]. Among the various technologies geared toward the treatment of organic dye wastewater, photocatalysis, sonocatalysis, and sonophotocatalysis have received increasing attention owing to their high efficiency and potential for large-scale industrial applications [9,10,11,12,13].

Photocatalysis is a well-established technology for the treatment of organic pollutants in wastewater [14,15,16,17]. Qutub et al. investigated CdS/TiO_2_ nanocomposites for photocatalytic degradation of organic pollutants in wastewater [18]. The results showed that CdS-TiO_2_ nanocomposites exhibited the highest photocatalytic activity in the degradation of AB-29 dye, with a degradation efficiency of 84%, compared to 68% and 9% achieved by CdS and TiO_2_ under comparable conditions, respectively. The enhanced photocatalytic performance of CdS-TiO_2_ was attributed to reduced charge carrier recombination, improved charge separation, and expansion of the response of TiO_2_ to visible light. In photocatalytic systems, semiconductor photocatalysts, such as TiO_2_, are irradiated with light, generating electron-hole pairs that react with water or oxygen to form reactive species, e.g., hydroxyl radicals. These reactive species can later degrade the organic pollutants into harmless products [19,20,21,22]. Sonocatalysis, on the other hand, utilizes ultrasonic wave to generate cavitation bubbles in the solution, which collapse and produce high-energy conditions that can promote chemical reactions. Wang et al. developed a recyclable WO_3_/NiFe_2_O_4_/BiOBr (WNB) composite with dual Z-scheme heterojunction for the degradation of levofloxacin (LEV) in aqueous solution [23]. The WNB composite showed the highest removal efficiency (97.97%) for LEV within 75 min under ultrasonic irradiation. The ternary composite comprises three different semiconductors suitable for harvesting full-spectrum light. The combination of sonocatalysis and photocatalysis, known as sonophotocatalysis, can further improve the efficiency of both processes, as the cavitation bubbles can create local “hot spots” that increase the photocatalytic activity for the catalyst [24,25,26,27,28]. Despite the promising results of sonophotocatalysis in pollutant degradation, there are still significant challenges that need to be addressed in order to enhance its performance for the treatment of organic dye wastewater [29]. For example, the high energy requirement of sonophotocatalysis limits its practical application, as it has high electricity consumption for generating the ultrasonic wave and producing the light [30,31,32,33,34,35,36]. In addition, mechanistic understanding on the synergistic effect of ultrasound and light in sonophotocatalysis is still not lacking, and more studies are required to clarify the underlying mechanism for optimization of the process [37,38]. Wang et al. synthesized Fe_3_O_4_@SiO_2_/PAEDTC@MIL-101(Fe), a mesoporous composite with a core-shell structure, and evaluated its sonophotocatalytic performance in degrading acid red 14 (AR14) [39]. The results showed that Fe_3_O_4_@SiO_2_/PAEDTC@MIL-101 (Fe)/UV/US exhibited excellent activity in the removal of AR14 and total organic carbon.

In this review, we aim to present an overview on the mechanisms of photocatalysis, sonocatalysis, and sonophotocatalysis in the treatment of organic dye wastewater [40,41]. We compare the performance of TiO_2_-based catalysts in photocatalytic, sonocatalytic, and sonophotocatalytic systems, with a focus on the synergistic effect of ultrasound and light in the catalytic degradation of organic dyes [42]. We further discuss the recent advances in catalyst development for sonophotocatalysis and point out the potential risks associated with the sonophotocatalytic process, such as the generation of toxic byproducts and the potential releases of nanoparticles into the environment [43,44,45,46,47,48]. It is essential to monitor the reaction products and assess their toxicity to ensure that the sonophotocatalytic process is safe for both the environment and human health. Finally, we propose future research directions to expand the fundamental knowledge on the sonophotocatalysis process and enhance its practical application in degrading organic pollutants [49,50,51,52,53]. The information presented in this review can provide valuable insights into the mechanisms and performance of photocatalysis, sonocatalysis, and sonophotocatalysis, and contribute to the development of more efficient and cost-effective treatment methods for organic dye wastewater. This review will be of great interest to researchers and practitioners in the field of environmental science and engineering, especially those involved in the development of sustainable wastewater treatment technologies.

## 2. Sonocatalytic and Photocatalytic Mechanisms

### 2.1. Sonocatalytic Mechanism

**Figure 1:**
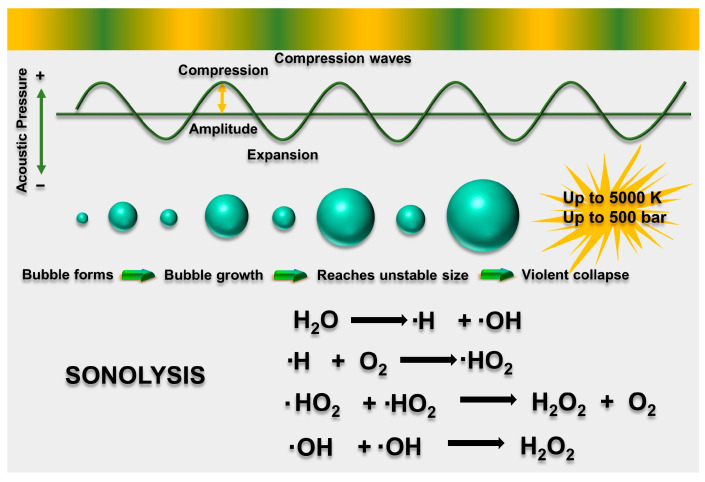
Schematic illustration of the acoustic generation of a cavitation bubble in water (after [66]).

The sonocatalytic process is believed to be predominantly based on the “hot spots” and “sonoluminescence” that originate from the ultrasonic cavitation phenomenon [54]. Ultrasonic wave of a specific frequency and intensity can produce numerous small bubbles in liquids [55]. These minute bubbles trigger various physical and chemical transformations during their formation, oscillation, expansion, contraction, and ultimate collapse [56,57,58]. Figure 1 scehematically depicts the phenomenon of ultrasonic cavitation. It promotes the production of light with a range of wavelengths, called “sonoluminescence,” and a large number of localized “hot spots” with very high temperatures (up to ~5000 K) and pressures (up to ~1000 atm) [59,60]. These localized “hot spots” can cause pyrolysis of H_2_O molecules, producing hydroxyl radicals (•OH) [61], which can effectively oxidize organic pollutants and even mineralize them into CO_2_ and H_2_O [62,63,64,65,66].

In general, the sonolytic removal of organic pollutants involves oxidation through both pyrolysis and free radical attack [67]. However, due to the significant energy loss that occurs during thermal dissipation (exceeding 50%), radpid degradation often cannot occur when relying solely on ultrasound [68]. In recent years, the use of ultrasound in the presence of appropriate catalysts, known as sonocatalysis, has been increasingly used to degrade organic pollutants due to its numerous benefits, including convenient handling and low cost, as well as environmental friendliness [69]. Sonocatalytic degradation involves the use of a sonocatalyst to create additional active sites for the cavitation effect, leading to the formation of greater numbers with highly reactive radicals [70]. In general, these radicals could recombine to form H_2_O, •OH, H_2_O_2_, and •O_2_^−^ in water [71,72]:

H_2_O + ))) → •H + •OH (1)

•H + •OH → H_2_O_2_ (2)

O_2_ + •H → •HO_2_ (3)

2•OH → H_2_O_2_ (4)

2•HO_2_ → O_2_ + H_2_O_2_ (5)

H_2_O + •OH → H_2_O_2_ + •H (6)

**Figure 2:**
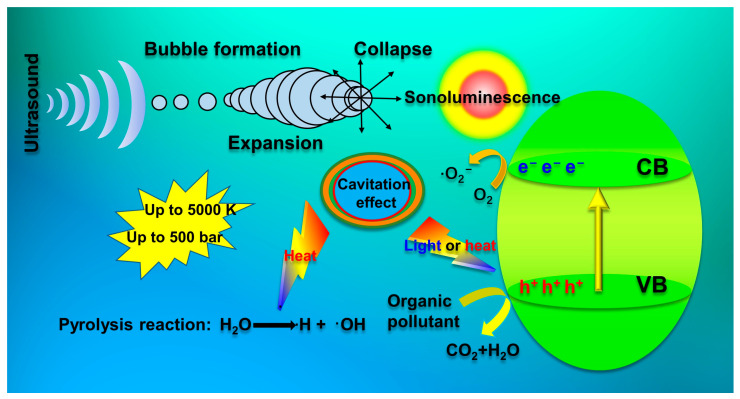
Schematic diagram of sonocatalytic mechanism (after [23]).

Sonocatalysis is a crucial technology in the degradation of pollutants, and radicals play an essential role in this process [73]. These radicals can initiate chain reactions that lead to the degradation of pollutants. To enhance the efficiency of the sonocatalytic process, it is essential to understand the underlying mechanisms of sonocatalysis [74]. Figure 2 depicts the major processes involved in sonocatalysis elucidated by extensive studies conducted in the field [75,76].

#### 2.1.1. Heterogeneous Nucleation Mechanism

Semiconductor particles have been observed to induce preferential formation of nuclei at solid surfaces or phase boundaries, leading to increased formation of cavitation bubbles and free radicals, such as •OH [54]. The phenomenon of heterogeneous nucleation has been found to be more applicable than homogeneous cavitation in sonocatalysis [56]. This can be attributed to the fact that the thermodynamic nucleation barriers on interfaces are generally lower than their bulk counterparts, promoting surface nucleation [18]. The relationship between the maximum energy barriers of heterogeneous and homogeneous nucleation processes can be expressed as [57,60]:

(7) $$\Delta {G^{*}}_{\mathrm{het}}=\frac{16{\pi \sigma }^{3}}{3P^{2}}\;f(\theta )=\Delta {G^{*}}_{\mathrm{hom}}\;f(\theta )\;$$

where ΔG_het_ and ΔG_hom_ are the maximum energy barrier for heterogeneous and homogeneous reactions, respectively, *σ* is the surface tension of water (J/m^2^), θ is the contact angle between the liquid and solid, and P is the sum of the partial pressure of the entrapped gas [47].

It is expected that preferential nucleation will transpire on hydrophobic surfaces, notably on solid surfaces [63]. Furthermore, the rate of bubble nucleation at the solid surface can be significantly influenced by many factors. Sonication parameters, such as ultrasonic power, frequency, as well as changes in surface energy, aqueous temperature, and type of absorbed gas, can greatly affect this process [59]. In addition, physicochemical properties of the solid particles, such as roughness, particle size, pore size, and wettability, can play a crucial role in influencing the nucleation rate.

#### 2.1.2. Photo-Excitation Mechanism

Sonoluminescence (SL) is a light-emitting phenomenon caused by the collapse of cavitational bubbles. The light emitted during sonoluminescence has high intensity and covers a wide range of wavelengths, typically between 200 and 700 nm [77]. In the presence of a semiconductor catalyst during ultrasonication, the energy from the light generated can exceed the band gap of the semiconductor, leading to the excitation of electrons from the valence band (VB) to the conduction band (CB) [78]. This process generates holes in the valence band, which are caused by the excited electrons [79]. When photogenerated electron-hole pairs react with dissolved oxygen, they create highly reactive radicals. This process in sonocatalysis is akin to that in photocatalysis [80].

#### 2.1.3. Thermal Excitation Mechanism

The “hot spots” hypothesis proposes that elevated temperatures in a specific area may result in thermal excitation of the semiconductor, causing the formation of electron-hole pairs [81]. This phenomenon has been observed in numerous studies [82], demonstrating that certain semiconductors can be stimulated by high temperatures to generate electron-hole pairs. At room temperature, TiO_2_ displays low catalytic activity, but its performance improves significantly after being heated to temperatures ranging between 350 and 500 °C [83]. Such enhancement is attributed to the abundant highly oxidative holes that arise due to the thermal excitation of semiconductors [84].

### 2.2. Photocatalytic Mechanism

**Figure 3:**
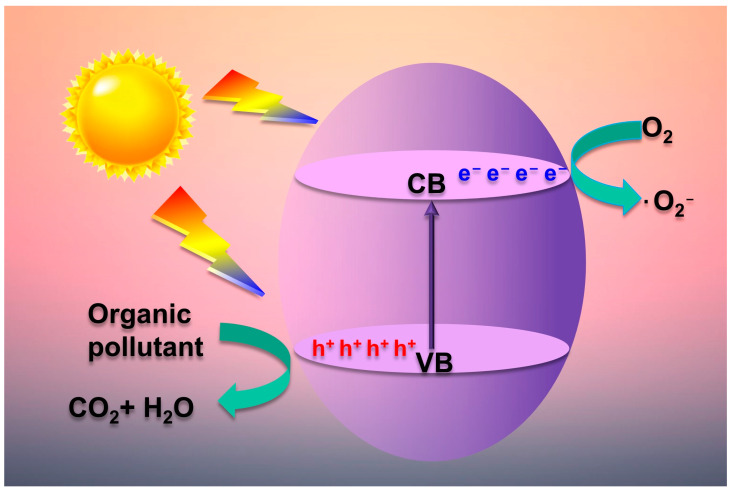
Schematic illustration of photocatalytic mechanism (after [23]).

The photocatalytic process occurs when a semiconductor catalyst is exposed to light of greater energy than the semiconductor’s bandgap [85], as depicted in Figure 3. When this happens, electrons in the VB may become excited and jump into the CB, forming a hole (h^+^_VB_) (Equation (8)). Subsequently, the electron-hole pairs that are generated by the absorption of light recombine together, leading to the emission of energy (Equation (9)) [86]. The poor quantum efficiency of the semiconductor is attributed to this recombination, which leads to low light-to-energy conversion rates [87,88]. If the photogenerated carriers do not recombine, light-generated electron-hole (e^−^, h^+^) pairs separate and move to the material’s surface, reacting with the adsorbed molecules [89]. When photo-excited electrons come into contact with dissolved oxygen molecules (O_2_) in an aqueous solution, they can react and form superoxide radical anions (•O_2_^−^), as indicated by Equation (10) [90]. At the same time, the holes may directly oxidize pollutants or H_2_O molecules to produce hydroxyl radicals (•OH) (Equation (11)). The reactive radicals generated (•OH, •O_2_^−^) are highly reactive oxidizing agents [91], andthey may readily mineralize many organic molecules, producing water and carbon dioxide (Equations (12) and (13)).

Semiconductor + hv → e^−^_CB_ + h^+^_VB_ (8)

e^−^_CB_ + h^+^_VB_ → energy (9)

e^−^ + O_2_ → •O_2_^−^ (10)

h^+^ + H_2_O → •H + •OH (11)

O_2_^−^ + Pollutant → H_2_O + CO_2_ (12)

•OH + Pollutant → H_2_O + CO_2_ (13)

### 2.3. Comparison of Sonocatalytic and Photocatalytic Mechanisms

Comparison of the mechanisms of sonocatalysis and photocatalysis can help better understand the unique features and advantages of sonocatalysis in promoting efficient and sustainable chemical transformations. The similarity and difference between these two types of mechanisms are detailed below.

#### 2.3.1. Similarity

Semiconductor catalyst plays a vital role in lowering the energy barrier for the formation of cavitation bubbles, which is similar to the way that a traditional catalyst reduces the activation energy of a chemical reaction [92]. This is achieved by providing a surface for the accumulation and stabilization of gas or vapor pockets within the fluid medium, effectively reducing the threshold pressure required for bubble nucleation [93].

Undoubtedly, photocatalysts have the potential to serve as effective sonocatalysts, leveraging the phenomenon of sonoluminescence generated by cavitation [94]. Given their inherent properties and unique chemical compositions, photocatalysts can harness the energy released by cavitation bubbles to enhance catalytic reactions and promote efficient chemical transformations [95].

#### 2.3.2. Difference

The formation of cavitation bubbles is primarily driven by physical processes, involving the rapid formation and collapse of small pockets of gas or vapor within a fluid medium [96]. This can occur due to the changes in pressure and temperature that cause the fluid to reach its boiling point, resulting in the generation of these bubbles [97]. The effect of cavitation can be significant, leading to the erosion of solid surfaces and the generation of shockwave that can have profound impacts on the surrounding environment [98].

Acoustic cavitation is a key phenomenon in sonocatalysis, whereby high-intensity sound wave generates microscopic bubbles in a liquid medium [99]. During the cavitation process, these bubbles release energy in the form of heat, shockwave, and free radicals, which can induce chemical reactions in the solution [100]. As the bubbles collapse, they generate extremely high temperatures and pressures in localized regions of the solution [101]. The sudden and intense energy release can result in large increases in temperature, which can accelerate the rate of chemical reactions in the solution [102]. Moreover, the high temperatures generated by acoustic cavitation can lead to thermal excitation of the catalyst, thereby promoting the generation of reactive species, such as electron-hole pairs [103]. This, in turn, can lead to enhanced catalytic activity and selectivity in sonocatalysis.

## 3. Sonophotocatalytic Process

### 3.1. Sonophotocatalytic Mechanism

**Figure 4:**
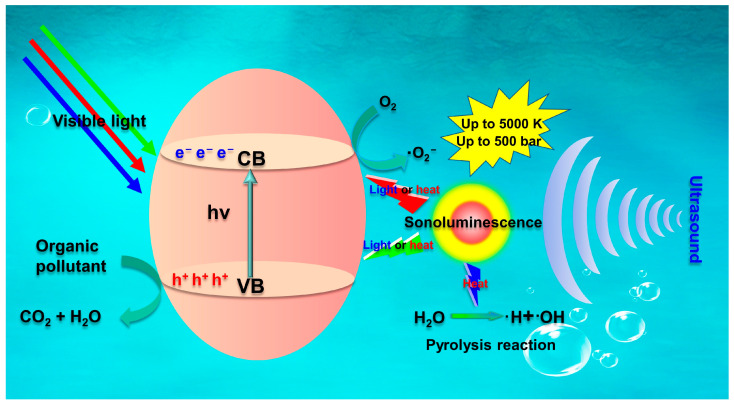
Schematic diagram of sonophotocatalytic mechanism (after [23]).

Sonophotocatalysis is essentially a combination of light, ultrasound, and catalyst that accelerates the degradation rates of organic pollutants via increasing the production of active radicals [104,105,106]. The highly efficient degradation of organic pollutants in sonophotocatalytic process is principally based on the synergistic effect of sonocatalysis and photocatalysis [107,108]. Figure 4 depicts the mechanism for the synergistic effect of photocatalysis and sonocatalysis. The key advantage of combining these two technologies is the greater number of cavitation bubbles generated via ultrasound, and more radicals generated via electron-hole pair separation in semiconductor photocatalysts. In addition, ultrasound continuously cleans the surface of the photocatalyst, which helps maintain the catalyst activity for extended periods. The combination of these two technologies can degrade hydrophobic and hydrophilic organic pollutants [109].

The sonophotocatalytic degradation of organic pollutants include the phenomena of both sonocatalysis and photocatalysis. Upon irradiation of ultrasonic wave, sonoluminescence and “hot spot” are generated due to cavitation in the aqueous solution. Moreover, the “hot spots” formed through ultrasonic cavitation may cause the pyrolysis of water molecules in contact with the surface of the sonocatalyst, generating hydroxyl radicals (•OH) and hydrogen radicals (•H) (Equations (14)–(19)) [110]. Subsequently, the light generated with a wide range of wavelength from sonoluminescence can excite the catalyst, facilitating charge carriers’ formation and the generation of electron-hole pairs in the CB and VB (Equation (20)). Additionally, irradiation of the catalyst’s surface with light increases the generation of electron-hole pairs and active radical species during sonophotocatalysis (Equation (21)) [111,112,113]. In the VB, holes react with water molecules adsorbed on the catalyst surface to generate •OH (Equation (22)). At the same time, electrons generated in the CB react with dissolved oxygen to generate •O_2_^−^, •OH, and H_2_O_2_ (Equations (23)–(25)). Subsequently, these active species react with organic pollutants to generate different degradation intermediates and even mineralization products (i.e., H_2_O and CO_2_) (Equation (26)) [114].

H_2_O + heat (hot spot) → •OH + •H (14)

H_2_O + H• → H_2_ +•OH (15)

•OH + •OH → H_2_O_2_ (16)

O_2_ + H• → HO_2_ (17)

HO_2_ + HO_2_ → O_2_ + H_2_O_2_ (18)

H_2_O_2_ + heat (hot spot) → 2•OH (19)

Semiconductor Catalyst + US → h^+^/VB + e^−^/CB (20)

Semiconductor Catalyst + hν → h^+^/VB + e^−^/CB (21)

h^+^/VB + H_2_O → •OH (22)

e^−^/CB + O_2_ → •O_2_^−^ (23)

2H_2_O + 2•O_2_^−^ → 2OH- + H_2_O_2_ + O_2_ (24)

2•O_2_^−^ + H_2_O_2_ → OH- + •OH + O_2_ (25)

Organic pollutants + Reactive oxidative species →H_2_O + CO_2_ (26)

### 3.2. Summary of the Synergistic Effect during Sonophotocatalytic Process

In order to compare the effects of sonophotocatalysis with those of separate processes (sonocatalysis and photocatalysis), it is necessary to assess the synergistic contribution to the elimination of organic pollutants during the degradation process by sonophotocatalysis. The synergistic effect of a sonophotocatalysis process can be assessed using the synergistic index. This index is calculated as the ratio of the rate constant of sonophotocatalysis to the sum of the rate constants of the individual processes, and is commonly employed to analyze the degree of synergistic enhancement in dye decolorization. The generic expression of the synergy index can be expressed as ([115,116]):

(27) $$\mathrm{Synergy}\;\mathrm{Index}=\frac{k_{\mathrm{sonophotocatalysis}}}{k_{\mathrm{sonocatalysis}}+k_{\mathrm{photocatalysis}}}$$

where k represents the pseudo-first-order rate constants of the photocatalytic, sonophotocatalytic, and sonocatalytic degradation processes, a synergistic index value of >1 means the efficiency of the sonophotocatalytic degradation is higher than the cumulative value of the individual processes (sonocatalytic or photocatalytic).

The synergistic effect of sonophotocatalysis in organic pollutant degradation has been demonstrated in many studies. Mosleh et al. reported that the pseudo-first-order rate constant for sonophotocatalytic degradation of trypan blue was 26.33 × 10^−2^ min^−1^, while the sum of the rate constants of photocatalysis and sonocatalysis was only 9.88 × 10^−2^ min^−1^, resulting in a synergistic index of 2.53 [117]. Babu et al. reported a synergistic index of 3.7 for the sonophotocatalytic degradation of Methyl orange using CuO-TiO_2_/rGO nanocatalysts [118]. The authors concluded that the high synergy probably resulted from the combined action of hydroxyl radicals generated by the sonolytic and photocatalytic systems. Benomara et al. reported that the pseudo-first-order rate constants for the degradation of methyl violet 2B were 6.8 × 10^−3^ for sonocatalysis, 22.9 × 10^−3^ for photocatalysis, and 39.7 × 10^−3^ min^−1^ for sonophotocatalysis, demonstrating the significant synergistic effect of sonophotocatalysis [119]. Ahmad et al. investigated the degradation of Rhodamine B (RhB) in photocatalytic, sonocatalytic, and sonophotocatalytic systems, and found that the sonophotocatalytic process exhibiting a higher rate constant compared to the sum of the photocatalytic and sonocatalytic processes [120]. Sonophotocatalytic process was more effective in degrading RhB compared to photocatalytic and sonocatalytic processes due to the presence of more reactive radicals and the increased active surface area of the ZnO/CNT photocatalyst. Togther, these findings highlight the potential of sonophotocatalysis as a promising approach for the efficient degradation of organic dyes in wastewater.

During the sonophotocatalytic process, the combination of ultrasonic wave, light, and photocatalyst can lead to synergistic effect that enhances the degradation of organic pollutants in wastewater. The synergistic effect is attributed to several factors, including the increased production of reactive radicals and the improved mass transfer of the pollutants to the photocatalyst surface. One of the key advantages of sonophotocatalysis is the increased production of reactive radicals, such as •OH, which is highly effective in breaking down organic pollutants. Ultrasonic wave can induce cavitation, which generates high-energy bubbles that collapse and release shockwave and heat, leading to the formation of reactive radicals. Similarly, when a photocatalyst is illuminated with light, electrons are excited, leading to the production of reactive radicals. The combination of ultrasonic wave and light in sonophotocatalysis can lead to a higher production of reactive radicals, as the ultrasonic wave can promote the separation of electron-hole pairs, which are the precursors of reactive radicals, while also enhancing the mass transfer of the pollutants to the photocatalyst surface. Another factor that contributes to the synergistic effect of sonophotocatalysis is the improved mass transfer of the pollutants to the photocatalyst surface. In traditional photocatalysis, the efficiency of pollutant degradation is often limited by the mass transfer of the pollutants from the bulk solution to the photocatalyst surface. The use of ultrasonic wave in sonophotocatalysis can enhance the mass transfer of the pollutants by promoting the formation of micro-scale streams and turbulence, which increase the contact between the pollutants and the photocatalyst surface. In summary, the synergistic effect of sonophotocatalysis in the degradation of organic pollutants can be attributed to the increased production of reactive radicals and the improved mass transfer of the pollutants to the photocatalyst surface.

## 4. Degradation of Dyes Using TiO_2_-Based Semiconductor Catalysts

TiO_2_ has been widely examined among numerous photocatalysts because of its chemical stability, non-toxicity, strong oxidation ability, low cost, high catalytic activity, and photo corrosion resistance. It has been the focus of research in the field of photocatalysis and is one of the most commonly used and most promising photocatalysts [121,122,123]. The photocatalytic activity of anatase TiO_2_ is limited to ultraviolet light with wavelength shorter than 387 nm due to its wide band gap of 3.23 eV. As the energy of UV light accounts for only 4% of the total energy of sunlight, TiO_2_ cannot effieiciently utilize sunlight, which seriously limits its application in photocatalysis [18]. In practical applications, researchers have modified TiO_2_ to enhance its catalytic activity. There are several primary methods for TiO_2_ modification, such as noble metal deposition, compound semiconductor, ion doping, and photosensitization. The primary objective of modification is to expand the light-absorption of TiO_2_ to the visible light spectrum and inhibit the recombination of electron-hole pairs [124]. Additionally, the incorporation of other materials into the TiO_2_ photocatalyst can enhance its performance. For example, graphene oxide (GO) has been used as a support material for TiO_2_ nanoparticles to form GO-TiO_2_ composites. The incorporation of GO can improve the adsorption capacity and photocatalytic activity of TiO_2_ by increasing the specific surface area and promoting the separation of photogenerated electron-hole pairs [125]. GO also has excellent electrical conductivity, which can facilitate the transfer of electrons and improve the efficiency of photocatalytic reactions. Moreover, metal ions, such as Fe, Cu, and Ag, can be doped into the TiO_2_ lattice to form metal-doped TiO_2_ photocatalysts. The incorporation of metal ions can modify the band gap of TiO_2_ and enhance its photocatalytic activity [126,127,128]. The metal ions can also act as active sites for the adsorption and degradation of organic dyes [129]. Therefore, the combination of TiO_2_ with other materials can enhance the its photocatalystic performance and broaden its application in the treatment of organic dye wastewater.

TiO_2_-based catalysts have shown promising performance in the degradation of organic dyes in various processes, including photocatalysis, sonocatalysis, and sonophotocatalysis. The efficiency of these processes depends largely on the generation of free radicals, such as •OH, •O_2_^−^. Table 1 summarizes the performance of TiO_2_-based catalysts in the degradation of organic dyes in recent studies.

Nuengmatcha et al. showed that the ZnO/graphene/TiO_2_ hybrid catalyst prepared using solvothermal method was more efficient at degrading ZGT dye compared to the indiviudal components [130]. The high surface area of graphene allows for better dispersion of ZnO and TiO_2_, leading to increased absorption of ultrasonic irradiation and the generation of more electron-hole pairs. Yao et al. synthesized TiO_2_/BiOBr heterojunctions with N/Ti^3+^ co-doping using one-step in situ hydrothermal method and demonstrated that they exhibited higher sonocatalytic activity in degrading methylene blue compared to pristine TiO_2_ [131]. Specifically, NT-TB_0.3_ exhibited the highest degradation efficiency of 98.2% after 50 min of ultrasound irradiation. The improved catalytic activity was attributed to the formation of a heterojunction between TiO_2_/BiOBr, which enhances the separation of electron-hole pairs. These studies highlight the potential of hybrid and composite catalysts in enhancing the performance of sonocatalytic and photocatalytic reactions and provide insight into the mechanisms underlying their improved activity.

Sriramoju et al. synthesized RGO-TiO_2−x_ nanocomposites using one-step in situ hydrothermal method and observed that these nanocomposites displayed exceptional photocatalytic degradation performance against diverse organic dyes when exposed to UV-visible irradiation [132]. The rate constants for Rhodamine-B, methylene blue, and rose red dye were 0.083 min^−1^, 0.075 min^−1^, and 0.093 min^−1^, respectively. The superior photocatalytic performance observed in TiO_2−x_ samples was linked to the presence of highly conductive RGO, which improves the mobility of photo-generated charge carriers and reduces electron-hole pair recombination. The oxygen vacancy/Ti^3+^ was also identified as an important contributor to the enhanced photocatalytic activity. Mousavi and colleagues developed a Z-scheme heterojunction photocatalyst consisting of Black-TiO_2_ and CoTiO_3_, which exhibits visible-light responsiveness and is capable of decomposing a variety of organic dyes, includimg rhodamine B, methylene blue, and methyl orange [133]. The much higher photocatalytic activity of B-TiO_2_/CTO nanocomposites compared to B-TiO_2_ and CTO is attributed to the improved generation, separation, and transportation of charge carriers. Moreover, the combination of B-TiO_2_ with CTO increased the specific surface area of the nanocomposites, which increases the active sites on the catalyst surface and the generation of photo-generated electron-hole pairs. These studies highlight the potential of constructing hybrid nanocomposites of TiO_2_ to enhance the photocatalytic removal of organic dyes.

Lozano et al. synthesized a novel Ag-graphene oxide/TiO_2_ catalyst and showed that it effectively degraded Black 5 and orange II dyes in a sonophotocatalysis system under ultrasonic and UV irradiation [134]. Ultrasound and UV light were observed to have significant synergistic effect on the degradation of organic dyes in the presence of the catalyst. Sun et al. investigated the sonophotocatalytic removal of organic pollutants in water using N/Ti^3+^-doped biphasic TiO_2_/Bi_2_WO_6_ heterojunctions, and found that the catalytic activity of NT-TBWx in the sonophotocatalytic system for the degradation of methyl blue was much higher than that in the photocatalytic and sonocatalytic systems [135]. Compared to TiO_2_ and NT-TiO_2_, the NT-TBWx heterojunctions exhibited superior sonophotocatalytic activity. The improved sonophotocatalytic efficiency of the NT-TBWx composites is likely due to the synergistic effect of photocatalysis and sonocatalysis, as well as the N/Ti^3+^ co-doping and heterophase junctions.

The performance of sonocatalytic, photocatalytic, and sonophotocatalytic processes is influenced by a range of factors, including catalyst dose, solution pH, and the type and concentration of organic dyes. These factors must be optimized to improve the overall dye removal efficiency in practical applications. For instance, pH plays a critical role in determining the surface charge potential of the catalyst, which can significantly impact its interaction with the organic dye molecules. Under acidic conditions, the surface of TiO_2_ is positively charged, allowing the adsorption of negatively charged dye molecules, thus increases the efficiency of the photocatalytic degradation. On the other hand, the surface of TiO_2_ becomes negatively charged under alkaline conditions, which reduces photocatalytic activityin the degradation of negatively charged dyes. The catalyst dose is another crucial factor that affects the performance of TiO_2_-based catalysts. The amount of catalyst used affects the number of active sites available for the adsorption of dye molecules, which directly influences the overal degradation rate. However, high catalyst doses may cause light shielding in the solution and reduce photocatalytic performance. In addition, the type and concentration of organic dyes also play important roles in the efficiency of TiO_2_-based catalysts. The adsorption of dye molecules on the surface of TiO_2_ is influenced by the size, structure, and chemical composition of the dye molecules, which affect the overall degradation rate. High concentrations of organic dyes can lead to increased light scattering and lower photocatalytic activity.

Taken together, photocatalytic, sonocatalytic, and sonophotocatalytic activity of TiO_2_-based catalysts is influenced by various factors, such as pH, catalyst dose, and the type and concentration of organic dyes. In practical applications, it is necessary to optimize these factors to achieve efficient treatment of organic pollutants in water and wastewater. Addressing the challenges of high cost and limited efficacy under visible light is crucial for the widespread adoption of TiO_2_-based catalysts in water and wastewater treatment.

## 5. Further Research Trends

While the general mechanism of sonophotocatalysis has been relativly well understood, there are several challenges that need to be addressed to make it a practical and effective method for dye decolorization. One of the major challenges is the scale-up of the sonophotocatalytic process from laboratory to industrial scale, as the reaction conditions and equipment used in the laboratory may not be suitable for large-scale applications. Thus, it is necessary to develop and optimize the sonophotocatalytic process for industrial applications, which may require innovative catalyst and reactor design, as well as novel ways of supplying the ultrasound and light energy.

The cost-effectiveness of the sonophotocatalytic process is a crucial aspect that needs to be considered. Despite the tremendous photocatalytic activity of noble metal/TiO_2_ systems, their practical utilization is remarkably constrained due to the high cost as well as limited accessibility for precious metals. This hinders the widespread application of sonophotocatalysis for dye decolorization, necessitating the development of cost-effective catalysts with high activity and stability, such as non-noble metal-based catalysts or composites of TiO_2_ with other materials. To enhance the properties of TiO_2_-based heterojunction photocatalysts, more efficient synthesis techniques must be explored to produce catalysts with tailored morphologies and compositions. However, it is challenging to mass produce high-quality, homogeneous TiO_2_-based heterostructure photocatalysts. Therefore, the design as well as performance for TiO_2_-based heterojunctions must be further improved, which requires better understanding of the photocatalytic reaction mechanism. Additional investigation is required to explore both the thermodynamics and kinetics of surface catalytic processes, as well as the mechanism of charge carrier transfer. To enable the effective utilization of TiO_2_-based heterojunction photocatalysts in natural environments with sunlight, it is crucial to extend the excitation wavelength of photocatalysts, particularly by broadening their light-response from UV to visible light, which can enhance their solar conversion efficiency. Additionally, the effect of environmental factors, such as temperature, pH, and the presence of other pollutants, on the sonophotocatalytic process need to be investigated. Changes in these factors can affect the performance of sonophotocatalytic processes, necessitating the optimization of treatment conditions to aqequate decolorization efficiency. In brief, the cost-effectiveness, synthesis techniques, photocatalytic reaction mechanism, and environmental factors are critical aspects that must be considered to enhance the practical application of sonophotocatalytic processes for dye decolorization.

TiO_2_ is only responsive to UV light, which accounts for a small portion of the solar spectrum. Therefore, there is a need for the development of visible-light-responsive TiO_2_-based catalysts to expand their applications in water and wastewater treatment. Several strategies have been proposed to improve the visible-light responsiveness of TiO_2_-based catalysts, such as doping with transition metals, modifying with carbon materials, and forming heterojunctions with other semiconductors.

In addition to the technical challenges of sonophotocatalytic treatment of organic pollutants, more in-depth understanding of the mechanism of sonophotocatalytic degradation of dyes is essential. Investigating the interactions between the catalyst, organic dye, and environmental factors is critical for developing an effective and efficient sonophotocatalytic process. Characterization of the reaction pathways and intermediates of dyes enables the prediction of the toxicity and environmental impact of the degradation products. Therefore, future research should focus on developing detailed mechanistic models that can predict the reaction pathways and intermediates in sonophotocatalytic degradation of dyes. This requires a combination of experimental and theoretical approaches to elucidate the complex interplay between the catalyst, organic dye, and environmental factors.

Besides the above technical and mechanistic challenges for the sonophotocatalytic treatment of organic pollutants, there are potential risks associated with the process that must be assessed. The release of nanoparticles from catalyst breakdown may have adverse effects on human health and the ecosystem. This should be thoroughly explored, and appropriate measures should be taken to mitigate their impact on the ecosystem and human health. Nanoparticle release can be minimized by optimizing the sonophotocatalytic process and designing catalysts with minimal nanoparticle release. Overall, a comprehensive risk assessment of the sonophotocatalytic process is essential to ensure that it is safe and sustainable for practical applications.

## 6. Conclusions

The technology of sonophotocatalysis has become an important method for treating organic pollutants in water and wastewater. Sonophotocatalysis has significant synergistic effect, resulting in faster pollutant removal compared to sonocatalysis and photocatalysis. With significant improvements in terms of efficiency and treatment time, sonophotocatalysis has the potential to be a practical and effective method for dye decolorization,. However, several challenges need to be addressed, including scale-up, cost-effectiveness, optimization of process conditions, mechanistic understanding, and risk assessment, to ensure that sonophotocatalysis can be widely applied in the treatment of dye wastewater.

The synergistic effect of sonophotocatalysis offers unique opportunity to overcome some of the limitations of other treatment technologies, including sonocatalysis and photocatalysis. Therefore, further research in this field could lead to the development of new and efficient water treatment technologies that can address a wide range of environmental problems.

## Figures and Tables

**Table 1:** Summary of performance of TiO_2_ based catalysts in the degradation of organic dyes.

TiO_2_-Based Catalyst	Dye	Catalytic Conditions	Experiment Conditions	Result (Kinetic Constant (k) or Degradation Efficiency (%))	Ref.
ZnO/graphene/TiO_2_ (ZGT)	Methylene blue	Bath sonicator Power = 750 W Frequency = 20 kHz	[Catalyst] = 1.00 g/L [Pollutant] = 20 mg/L	1.97 × 10^−2^ min^−1^	[130]
N/Ti^3+^ TiO_2_/BiOBr0.3	Methylene blue, rhodamine B	Bath sonicator Power = 180 W Frequency = 30 kHz	[Catalyst] = 7.5 mg [Pollutant] = 5 mg/L Time = 50 min	98.2%	[131]
Er^3+^: YAlO_3_/TiO_2_-ZnO	Acid red B	Bath sonicator Power = 50 W Frequency = 40 kHz	[Catalyst] = 1.0 g/L [Pollutant] = 10 mg/L Time = 60 min	76.84%	[58]
RGO-TiO_2−x_	Methylene blue	Light power = 150 W	[Catalyst] = 20 mg [Pollutant] = 5 ppm	0.075 min^−1^	[132]
Black-TiO_2_/CoTiO_3_	Rhodamine B, methylene blue, and methyl orange	Light power = 50 W	[Catalyst] = 100 mg [Pollutant] = 5 ppm Time = 60 min	99%	[133]
Au-TiO_2_	Patent blue V	Light power = 570 W/m^2^	[Catalyst] = 23 g/L [Pollutant] = 7 mg/L Time = 180 min	93%	[88]
TiO_2__Ag_Graphene	Black 5	Bath sonicator Power = 30 W/L Frequency = 40 kHz UV light power = 5 W	[Catalyst] = 0.03 g [Pollutant] = 5 mg/L	0.05 min^−1^	[134]
NT-TBWx	Methylene blue (MB)	Bath sonicator Power = 180 W Frequency = 35 kHz UV light power = 100 mW/cm^2^	[Catalyst] = 7.5 mg [Pollutant] = 5 mg/L Time = 50 min	99%	[135]
CNTs/TiO_2_	methyl orange (MO)	Bath sonicator Power = 50 W Frequency = 20kHz UV light Power = 30 W	[Catalyst] = 50 mg [Pollutant] = 25 ppm	0.01118 min^−1^	[113]

## Data Availability

No data are associated with this article.
